# Blueberry Juice Antioxidants Protect Osteogenic Activity against Oxidative Stress and Improve Long-Term Activation of the Mineralization Process in Human Osteoblast-Like SaOS-2 Cells: Involvement of SIRT1

**DOI:** 10.3390/antiox9020125

**Published:** 2020-02-01

**Authors:** Vladana Domazetovic, Gemma Marcucci, Irene Falsetti, Anna Rita Bilia, Maria Teresa Vincenzini, Maria Luisa Brandi, Teresa Iantomasi

**Affiliations:** 1 Department of Biomedical Experimental and Clinical Sciences “Mario Serio”, University of Florence, Viale Morgagni 50, 50134 Florence, Italy; 2 Department of Chemistry “Ugo Schiff” University of Florence, Via U. Schiff, 6, 50019 Sesto Fiorentino, Italy

**Keywords:** blueberry juice (BJ), total soluble polyphenols (TSP), osteoporosis, dietary antioxidants, oxidative stress, osteoblast osteogenic differentiation, alkaline phosphatase (ALP), Runt-related transcription factor 2 (RUNX-2), sirtuin type 1 deacetylase (SIRT1)

## Abstract

Diets rich in fruits and vegetables with many antioxidants can be very important in the prevention and treatment of osteoporosis. Studies show that oxidative stress, often due to lack of antioxidants, is involved in alteration of bone remodeling and reduction in bone density. This study demonstrates in human osteoblast-like SaOS-2 cells that blueberry juice (BJ), containing 7.5 or 15 μg∙mL^−1^ total soluble polyphenols (TSP), is able to prevent the inhibition of osteogenic differentiation and the mineralization process due to oxidative stress induced by glutathione depletion. This situation mimics a metabolic condition of oxidative stress that may occur during estrogen deficiency. The effect of BJ phytochemicals occurs through redox- and non-redox-regulated mechanisms. BJ protects from oxidative damage factors related to bone remodeling and bone formation, such as alkaline phosphatase and Runt-related transcription factor 2. It upregulates these factors by activation of sirtuin type 1 deacetylase expression, a possible molecular target for anti-osteoporotic drugs. Quantitative analysis of TSP in BJ shows high levels of anthocyanins with high antioxidant capacity and bioavailability. These novel data may be important to elucidate the molecular and cellular beneficial effects of blueberry polyphenols on bone regeneration, and they suggest their use as a dietary supplement for osteoporosis prevention and therapies.

## 1. Introduction

Recent studies show that changes in the oxidative state and the regulation of redox homeostasis affect bone turnover and remodeling [1,2,3]. Excessive production of reactive oxygen species (ROS), not counterbalanced by endogenous antioxidant defense systems, induces oxidative stress with consequent abnormal osteocyte apoptosis, which activates the osteoclasts and inhibits osteoblast osteogenic activity [1,4,5,6]. This is related to estrogen deficiency, aging, or bone inflammatory processes in which oxidative stress induces low bone mineral density and loss of bone mass [3,7,8,9]. A few recent clinical studies showed that an imbalance at a cellular level between ROS and antioxidants seems to be involved in the pathophysiology of bone-related diseases [1,10,11,12]. Oxidative stress and a decreased reduced glutathione/oxidized glutathione (GSH/GSSG) ratio are associated with the inhibition of osteoblast differentiation and the mineralization process and alter the levels of specific osteogenic markers [13]; furthermore, the ROS increase activates osteoclast differentiation [10,14]. These events are often associated with bone metabolic diseases such as osteoporosis, the most common bone disease, in which oxidative stress is considered a significant risk factor for its development [7,8,9]. Increased ROS production is responsible for diverting bone precursor cell differentiation toward the formation of other cell types rather than bone tissue cells [15,16]. On the contrary, antioxidants counteract these negative effects and favor the activity of osteoblasts, the viability of bone stem cells, and the maintenance of a normal bone remodeling process [1,3,6,11,12,16].

Literature data obtained in animals or cell lines showed the antioxidant activity of various natural substances [1]. In particular, diets rich in vegetables and/or fruits with high content of active antioxidants, such as polyphenols including flavonols, isoflavones, and phytosterols/phytoestrogens, can have an important role in prevention and/or management of osteoporosis and bone inflammatory diseases related to oxidative stress [17,18,19,20,21]. Indeed, plasma antioxidant content was found diminished in osteoporotic women [11,22]. These antioxidant compounds scavenge ROS [1,6,16,23,24] and reduce oxidative stress in many diseases including bone diseases and, in particular, osteoporosis [17,22,24,25,26,27]. Many literature data suggest a positive relationship between antioxidant intake and prevention of bone loss often associated with increased bone fractures [18,20,24,27]. For this, osteoporosis is considered a highly debilitating and socially relevant pathology; in fact, among the elderly, the pathological consequences of osteoporosis are among the main causes of mortality [28,29]. Recently, it was shown that diets containing blueberry (BB) prevent osteoporosis in ovariectomized rats [19,26,30]; indeed, BB and, in particular, *Vaccinuim myrtillus* (VM) have a wide variety and high concentrations of well-characterized polyphenols such as anthocyanins, coumarins, flavonols, flavanols, and their phenolic derivatives [31,32], with beneficial properties in bone anabolism [17,18,19,20,21]. Moreover, recent studies suggest VM as a “functional food” and, as such, of benefit for dietary supplementation [31,32]; today, VM, together with *Vaccinium corymbosum*, is among the main species of BB used in the food industry. Recently, we demonstrated that blueberry juice (BJ), mainly obtained from VM, exhibits osteogenic action, through its antioxidant and antiosteoclastogenic effect, in murine osteocytes, MLO-Y4. Moreover, it shows a protective effect in bone marrow mesenchymal stromal cells (MSCs), fundamental for cell therapy in bone diseases, by preventing oxidative stress-induced toxicity [33].

The aim of this study was to evaluate the effect of BJ containing certain quantities of soluble polyphenols on the factors related to differentiation and the mineralization process of osteoblasts in the presence or absence of oxidative stress. In fact, the role of these dietary polyphenols on osteogenic activity of osteoblasts and on redox-regulated molecular processes involved in bone formation and regeneration is still little known. In particular, no data are reported on the molecular mechanisms involved in the protective action of BJ phytochemicals against oxidative stress-induced damage on osteogenic activity of osteoblasts. Moreover, it is interesting to assess effects of complex mixture of phytochemicals on osteoblast activity, considering that individuals consume fruit and vegetables rich in a variety of polyphenols. Indeed, some studies show that various polyphenols and their derivatives are bioavailable from BB and they are also absorbed in humans in intact form [34,35,36]. Finally, this study was performed to elucidate, at a cellular and molecular level, the beneficial effects of BJ polyphenols on bone regeneration, before suggesting their use as a dietary and pharmacological supplement for the prevention and/or management of osteoporosis and other bone diseases related to oxidative stress.

This study was performed in human osteoblast-like cell line SaOS-2 in which oxidative stress was induced by an intracellular depletion of GSH by butionine sulfoximine (BSO), a specific inhibitor of γ-glutamylcysteine synthetase that regulates GSH synthesis [13], before starting osteogenic differentiation and during the early phases of the mineralization process. We used SaOS-2 cells given that, in these cells, BSO-induced oxidative stress inhibits osteogenic factors involved in the final stage of osteoblast activity and related to differentiation and the mineralization process [13]. These cells reflect a normal phenotype of osteoblasts [37,38] and, like them, display the entire differentiation sequence and are able to form an extracellular mineralized matrix [39]. All these features contribute to the SaOS-2 cell line being considered as a cellular model to study osteoblast functions and, in particular, processes associated to late osteoblastic differentiation stage in human cells, such as the formation of bone nodules by differentiated osteoblasts [37,40,41]. 

## 2. Materials and Methods

### 2.1. Reagents

All common reagents were purchased from Sigma-Aldrich (Saint Louis, MO, USA), Extrasynthèse (Genay, France), GE Healthcare (Little Chalfont, Great Britain), Santa Cruz Biotechnologies (Santa Cruz, CA, USA), Millipore (Bedford, MA, USA), Abcam (Cambridge, UK), Euroclone (Milan, Italy), Thermo Scientific (Waltham, MA, USA), Bioassay Systems (Hayward, CA, USA), Promega (Madison, WI, USA), and Invitrogen (Carlsbad, CA, USA), unless differently specified in the text.

The following reagents were purchased from Sigma-Aldrich: Ham’s F12 Coon’s modification medium, l-glutamine, dimethyl sulfoxide (DMSO), BSO, trypsin, bovine serum albumin, Tris/HCl, Triton X100, NaCl, NaF, ethylene bis(oxyethylenenitrilo)tetraacetic acid (EGTA), β-glycerophosphate, human sirtuin type 1 (SIRT1) small interfering RNA (siRNA), Universal Negative Control #1, Alizarin Red S, phenolic reference standards ellagic acid (assay HPLC ≥ 95%) for hydroxybenzoic acids, (+)-catechin hydrate (assay HPLC ≥ 96.0%) for flavan-3-ols), acetonitrile HPLC grade (assay 99.9%), formic acid for (HPLC assay 98–100%), ascorbic acid, dexamethasone, paraformaldehyde, cetylpyridinium chloride, Folin–Ciocalteu reagent, NaCl/Pi.

The following reagents were purchased from Extrasynthèse: anthocyanin reference standard cyanidin 3-glucoside chloride (assay HPLC ≥ 96%), flavonol reference standard quercetin 3-*O*-glucoside (assay HPLC ≥ 99%), hydroxycinnamic acid reference standard 3-*O*-caffeoyl quinic acid (chlorogenic acid, assay HPLC ≥ 99%).

The following reagents were purchased from GE Healthcare: penicillin/streptomycin 100× solution, phosphate-buffered saline (PBS), polyvinylidene fluoride (PVDF) membrane, enhanced chemiluminescence (ECL) Western Blotting Detection Reagent kit. 

The following reagents were purchased from Santa Cruz Biotechnologies: EX527, Protein A/G PLUS-Agarose, anti-Runt-related transcription factor 2 (RUNX-2), anti-phospho-tyrosine, anti-histone H3.

The following reagents were purchased from Milipore: Milli-Q water, Cytobuster Protein Extraction Reagent.

The following reagents were purchased from Abcam: SIRT1 ELISA kit, anti-histone H3.

The following reagent was purchased from Euroclone: fetal bovine serum South American origin. 

The following reagent was purchased from Thermo Scientific: Pierce bicinchoninic acid (BCA) protein assay kit.

The following reagent was purchased from Bioassay Systems: QuantiFluo Alkaline Phosphatase Assay Kit.

The following reagent was purchased from Promega: CellTiter-Glo Luminescent Cell Viability Assay.

The following reagents were purchased from Invitrogen: lipofectamine RNAiMAX^TM^, 2′,7′-dichlorodihydrofluorescein diacetate.

### 2.2. Preparation of Blueberry Juice and Determination of Total Soluble Polyphenols

BBs, harvested in August 2018/2019 in Tuscany Apennines and supplied by IL BAGGIOLO S.R.L. (Abetone, Pt, Italy) and DANTI GIAMPIERO S.R.L. (Cutigliano, Pt, Italy), were frozen freshly picked in aliquots of 100 g each and homogenized in a refrigerated Waring Blender to prepare BJ. Insoluble particles were removed by filtration under vacuum and centrifuged at 20,000× *g* for 10 min. Aliquots of BJ were stored at −20 °C until use. The total soluble polyphenol (TSP) fraction of BJ was quantified with Folin–Ciocalteu reagent using gallic acid as the standard as described in our previous work [33] and via the HPLC method reported below. TSP concentration in BJ obtained from 100 g of BB fresh weight was expressed as mg/100 mL ± SD and the values measured by Folin–Ciocalteu assay or HPLC method were 169.5 ± 19.4 and 158.8 ± 12.3, respectively. 

### 2.3. HPLC-PDA-MS Analysis of Phenolic Compounds

The identification of phenolic compounds was performed using a Waters Alliance 2695 coupled online with a Waters 2996 photodiode array detector, and with a Quattro micro mass spectrometry detector with an electrospray interface. Separations were performed on a C18 reversed-phase Gemini Phenomenex (150 × 3 mm, 5 µm particle size) with a mobile phase flow rate of 0.4 mL∙min^−1^. The mobile phase consisted of (A) H_2_O containing 5% formic acid and (B) MeCN. A gradient elution program was applied as follows: 0–1.0 min held on 8% B, 1.0–16.0 min linear gradient to 15% B, 16.0–28.0 min linear gradient 50% B, 28.0–36.0 min linear gradient to 95% B, then in 1 min to the initial (starting) condition, and held 8 min for re-equilibration. The total run time was 45 min. The sample was diluted 1:10 (*v*/*v*) with 8% B and 92% A, with an injection volume of 10 µL. Determination of phenolic compounds was performed using two detectors online: a photodiode array UV detector, followed by a single quadrupole mass spectrometry detector. The photodiode array scanned the samples at λ_max_ 270, 320, 360, and 520 nm. The mass spectrometer detector was optimized to the following conditions: capillary voltage 3.20 kV, source block temperature 125 °C, and desolvation temperature 350 °C, operating in electrospray positive mode, detection range 100–1000 Da with total ion count extracting acquisition. The cone voltage was 32 V, the extractor lens was 3 V, and the cone and desolvation gas flows were 20 and 320 L∙h^−1^, respectively. Phenolic compound identification in the sample was carried out by comparing UV absorption spectra and mass spectra of each compound with those reported in the literature [42]. The quantification of polyphenols was calculated using the method of an external standard. Each standard was freshly prepared up to 300 μg/mL concentration and injected three times to obtain its calibration curve. Quantification was obtained as total content of each polyphenol group. Quantification of total constituents of each class of flavonoids was carried out using single anthocyanin, flavonol, and flavan-3-ol standards, namely, cyanidin-3-glucoside, quercetin-3-glucoside, and (+)-catechin equivalents, respectively. The values were expressed as gallic acid equivalent.

### 2.4. Cell Cultures, Treatments, Osteogenic Differentiation, and Cellular Viability

Osteoblast-like SaOS-2 cells were cultured in Ham’s F12 Coon’s modification medium, supplemented with 10% fetal bovine serum, 2 mM l-glutamine, 72 mg/L penicillin, and 100 mg/mL streptomycin (growth medium, GM), and incubated at 37 °C in a 5% CO_2_ humidified atmosphere with 20% oxygen. For the experiments of osteogenic differentiation, when SaOS-2 cells reached 70–80% confluence, GM was changed with osteogenic medium (OM) to induce osteogenic differentiation. OM was the growth medium supplemented with 10 nM dexamethasone, 0.2 mM ascorbic acid, and 10 mM β-glycerophosphate. Then, 40 μM BSO, or BJ containing 7.5 or 15 μg∙mL^−1^ TSP, or BJ + BSO, or BJ + 10 μM EX527, or BJ + BSO + 10 μM EX527 were added or not to GM for 24 h (day 1) before exchanging it for OM, containing or not the before mentioned compounds, to stimulate the differentiation process. The OM was refreshed twice a week for the whole study period, BSO was added for only two days after the beginning of differentiation and BJ or EX527 were added to the OM at each change from the beginning of the differentiation process for the whole study period.

Cell viability was evaluated using the CellTiter-Glo Luminescent Cell Viability Assay, according to the manufacturer’s instructions. 

In some experiments, the cells were transiently transfected with 75 nM human SIRT1 siRNA corresponding to two DNA target sequences of human SIRT1 (5′-GUGUCAUGGUUCCUUUGCA[dT][dT]-3′ accession number SASI_Hs01_00153666; 5′-UGCAAAGGAACCAUGACAC[dT][dT]-3′, accession number SASI_Hs01_00153666 6_AS) or scrambled siRNA (Scr siRNA) (Universal Negative Control #1), using lipofectamine RNAiMAX^TM^, according to the manufacturer’s instructions. The ability of SIRT1 siRNA to silence SIRT1 expression levels of about 50% was checked in control cells transfected for 24 h in GM and for other 48 h in OM. Additionally, 0.008% DMSO was present in experiments with EX527 in all conditions.

### 2.5. Determination of Intracellular ROS

The cell-permeant probe, 2′,7′-dichlorodihydrofluorescein diacetate (H_2_DCFDA), was added in the culture medium of SaOS-2 seeded in 12-well plates one hour before the end of the various treatments performed as written above. The probe after deacetylation by esterases is rapidly oxidized to a highly fluorescent compound in the presence of ROS. After PBS washing, adherent cells were lysed in radioimmunoprecipitation assay RIPA buffer (50 mM Tris/HCL pH 7.5, 1% Triton X-100, 150 mM NaCl, 100 mM NaF, 2 mM EGTA, phosphatase, and protease inhibitor cocktail), centrifuged at 20,000× *g* (ALC PK121R, Thermo Fisher Scientific, Waltham, MA, USA) for 10 min, and the intracellular levels of ROS were measured by florescence analysis at 510 nm. The normalization of the data was obtained by using total proteins, and the values were expressed as percentages with respect to the controls.

### 2.6. Alkaline Phosphatase Activity

SaOS-2 cells seeded in six-well plates during differentiation in the presence or not of the various treatments, as described above, were collected in Cytobuster Protein Extraction Reagent (Milipore, Burlington, MA, USA). After sonication twice on ice and centrifugation at 4 °C for 15 min at 1000× *g*, alkaline phosphatase (ALP) activity was measured in the supernatants using the QuantiFluo Alkaline Phosphatase Assay Kit following the manufacturer’s instructions. The ALP activity was normalized to protein content for each well, and data were expressed as percentages relative to the control values. 

### 2.7. Western Blot Analysis of RUNX and RUNX-2 Phosphorylation 

Western blot analysis was performed in SaOS-2 cells six days after differentiation and treated or not (control) as described above. Whole-cell lysates and nuclear extracts were obtained as previously described in References [13,33], respectively. Equal amounts of nuclear proteins were then incubated with antibody against Runt-related transcription factor 2 (RUNX-2) for 1 h at 4 °C. Subsequently, the immune complexes were precipitated using Protein A/G PLUS-Agarose. The immunoprecipitates (200 μg) were mixed with Laemmli buffer for 5 min at 95 °C, subjected to SDS/PAGE, and electrotransferred to a PVDF membrane [13]. Phospho-RUNX-2 (p-RUNX-2), RUNX-2, histone H3, and β-actin were visualized using antibody anti-phospho-tyrosine proteins, anti-RUNX-2, anti-histone H3, or anti-β-actin, respectively. Antigen–antibody complexes were detected using chemiluminescence ECL Western Blotting Detection Reagent kit. Digital images of the bands were detected by Amersham Imager A600 (GE Healthcare, Chicago, IL, USA).

### 2.8. Alizarin Red S Assay

The deposition of calcium was measured 12 and 24 days after differentiation in cells treated as described above. Cells were fixed in 4% paraformaldehyde for 15 min after washing twice with NaCl/Pi for a few minutes; subsequently, they were washed another three times with deionized water. Calcium mineral deposits were stained by using 2% Alizarin Red S at pH 7.8 for 2 min and were destained using 10% cetylpyridinium chloride in deionized water for 60 min at 50 °C. The absorbance of Alizarin Red S extracts was measured at 560 nm. Calcium content was evaluated using a standard curve of hydroxyapatite (100 μg/mL in cetylpyridinium chloride solution) and expressed as mg hydroxyapatite (HA) per cm^2^.

### 2.9. SIRT1 Expression Assay

SIRT1 levels were measured by using the Human SIRT1 ELISA kit in SaOS-2 cells, seeded in 12-well plates. Cell were solubilized in Cell Extraction Buffer and centrifuged at 18,000× *g* for 20 min at 4 °C according to manufacturer’s instructions. Data, normalized on total protein content, were expressed as percentages of control levels.

### 2.10. Protein Assay

Protein concentrations were determined by the bicinchoninic acid solution protein reagent assay using bovine serum albumin as the standard. 

### 2.11. Statistical Analysis

One-way ANOVA analysis with Bonferroni’s multiple comparison test, using GraphPad Prism Software, or Student’s *t*-test were used to determine the statistical significance. A *p*-value ≤ 0.05 was considered statistically significant.

## 3. Results

### 3.1. Polyphenolic Composition of BJ 

Phenolic constituents from BJ were identified by HPLC-PDA-MS analysis as reported in Section 2. The chromatographic profile displayed a suitable separation of all the polyphenol constituents in the juice after just one run, and the data analysis is reported in Table 1. 

Fourteen anthocyanins were identified using UV wavelength detection at 520 nm and mass spectra in the positive ion mode, since these compounds are present as flavylium ions in the chromatographic conditions applied. Delphinidin, cyanidin, petunidin, peonidin, and malvidin were the representative aglycons, glycosylated at C-3 with the same sugars of anthocyanins, namely, glucose, galactose, and arabinose. Similarly, five flavanols were identified using UV wavelength detection at 360 nm and mass spectra in both the positive and the negative ion mode, as reported in Table 1. Aglycons were identified as quercetin and myricetin glycosylated at C-3. Two further flavonoid structures were identified, the flavan-3ols catechin and epicatechin, using the same strategy reported for the other flavonoids. Finally, ellagic acid and chlorogenic acid were unambiguously identified as simple polyphenols, by comparison of their retention times and UV and mass spectra with those of the authentic samples. Table 1 shows the total content of each group expressed as gallic acid. Anthocyanins, expressed as cyanidin-3-glucoside, represented the main constituents corresponding to about 0.63 mmol/100 mL of juice, whereas flavonol and catechin contents were about 0.017 and 0.034 mmol/100 mL, expressed as quercetin-3-glucoside and (+)-catechin equivalents, respectively. The amount of ellagic acid was ca. 0.073 mmol/100 mL juice, while that of chlorogenic acid was ca. 0.18 mmol/100 mL. 

### 3.2. Effect of BJ on Cell Viability and in Preventing Oxidative Stress Induced by BSO Treatment in SaOS-2 Cells

Initially, we assessed cell viability during the pre-treatment (24 h in GM, day 1) before the induction of the differentiation and after two days of differentiation in the presence or not (control) of BSO and BJ (Table 2). 

In particular, BSO treatment was performed at the 40 μM concentration that induces oxidative stress due to significant decrease of GSH/GSSG ratio, an index of the intracellular oxidative status, as previously reported in SaOS-2 cells [13]. BJ containing two different concentrations of TSP (7.5 or 15 μg∙mL^−1^), which previously demonstrated antioxidant activity in starved osteocytes [33], was used. Table 2 shows that neither BSO nor the highest concentration of BJ, used alone or together with BSO, significantly altered cell viability as compared to control cells. 

**Figure 1:**
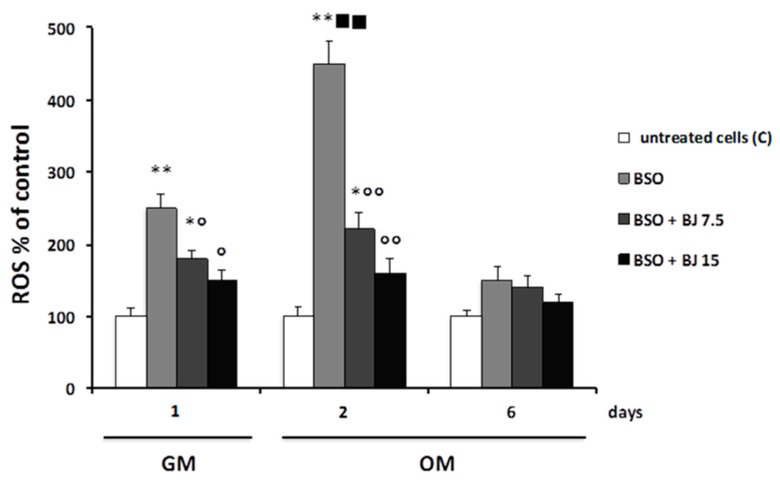
BJ effect on intracellular reactive oxygen species (ROS) production in BSO-treated SaOS-2 cells. Intracellular ROS, detected by measuring the fluorescence intensity of the probe 2′,7′-dichlorodihydrofluorescein diacetate (H_2_DCFDA), were measured in SaOS-2 cells treated or untreated (C; control) with BSO or BSO + BJ containing 7.5 or 15 μg·mL^−1^ total soluble polyphenols (TSP) for one day in growth medium (GM) and subsequently treated or untreated (C; control) for two or six days in osteogenic medium (OM), as reported in Section 2. ROS data, normalized on total protein content, are expressed as a percentage of the C values, and they are the means ± SEM of four experiments repeated in triplicate; * *p* ≤ 0.05, ** *p* ≤ 0.001 compared to C cells; ^○^ *p* ≤ 0.05, ^○○^ *p* ≤ 0.001 compared to BSO-treated cells; ^■■^ *p* ≤ 0.001 compared to BSO-treated cells for one day in GM.

Figure 1 reports the BJ antioxidant effect on BSO-treated cells. In fact, BSO pre-treatment (24 h in GM, day 1) was able to significantly increase ROS levels as compared with the control values measured in untreated cells (Figure 1). 

ROS levels increased further when BSO was subsequently added for other two days in OM as compared with control (Figure 1); from this time on (two days), BSO was no longer added, and ROS content returned to the control levels after six days after the induction of differentiation (Figure 1). In order to prevent the effect of BSO, the cells were treated simultaneously with BSO and BJ containing 7.5 or 15 μg∙mL^−1^ TSP. Figure 1 reports that BJ at both concentrations significantly prevented ROS increase in SaOS-2 cells after just 24 h in GM, and this effect was even more marked (by about 50–70%) after two days after the induction of differentiation. The highest concentration was able to decrease ROS levels to control values. No change in the intracellular oxidative state was observed after six days after the induction of differentiation in all conditions used (Figure 1). Similarly, no change in ROS levels was observed, when only BJ at both TSP concentrations was added before and during the differentiation at all studied times (data not shown), indicating that BJ per se does not have any effect on a normal cellular redox state. 

### 3.3. BJ Effect on the Markers of Differentiation and Osteogenic Process in SaOS-2 Cells in the Presence or Not of BSO-Induced Oxidative Stress

**Figure 2:**
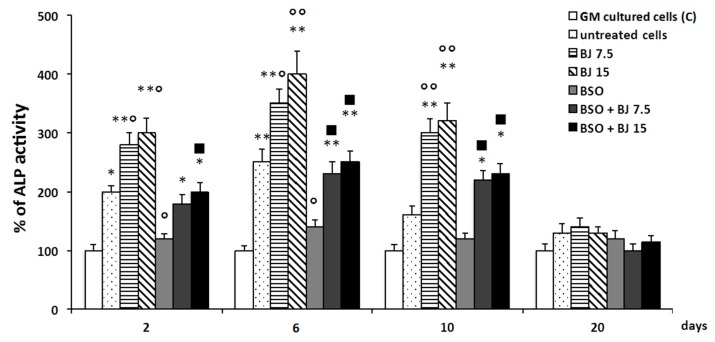
BJ effect on alkaline phosphatase (ALP) activity during differentiation of BSO-treated and untreated SaOS-2 cells. ALP activity, detected by the QuantiFluo Alkaline Phosphatase Assay Kit, was measured in SaOS-2 cells treated or untreated with BJ, containing 7.5 or 15 μg·mL^−1^ total soluble polyphenols (TSP), or BSO or BSO + BJ for one day in growth medium (GM). Subsequently, untreated cells were cultured in GM (C; control) for two, six, 10, or 20 days and, for the same times, treated and untreated cells were cultured in osteogenic medium (OM), as reported in Section 2**.** ALP activity is expressed as a percentage of the respective C values, and these are the means ± SEM of four experiments performed in triplicate; * *p* ≤ 0.05, ** *p* ≤0.001 compared to C cells; ^○^ *p* ≤ 0.05, ^○○^ *p* ≤ 0.001 compared to untreated cells; ^■^ *p* ≤ 0.05 compared to BSO-treated cells.

In SaOS-2 cells treated or not with BSO, BJ, or BSO + BJ, as reported in Section 2, the levels of alkaline phosphatase (ALP, EC 3.1.3.1), an early biochemical marker of osteoblast differentiation and osteogenic activity, were measured [13]. This enzyme is considered an osteoblast phenotype marker in SaOS-2 cells and, therefore, an osteoblast differentiation indicator [37,43]. Figure 2 shows the time course of ALP activity in SaOS-2 cells as a percentage of the activity values measured in cells cultured in GM (controls). 

ALP activity significantly increased in the early phase of differentiation process (2–6 days) in untreated cells cultured in OM as compared to the respective controls. Ten days after the induction of the differentiation, the percentage of ALP increase was no longer significantly different from the control (Figure 2). The ALP trend, in cells treated with BJ containing both concentrations of TSP, showed that its activity was higher during the initial phase of differentiation with respect to the untreated cells, and it remained high even after 10 days, unlike what happened in the untreated cells (Figure 2). It is worthy to note that the BJ effect occurred in cells with normal redox state. On the contrary, BSO significantly inhibited the upregulation of ALP activity levels (Figure 2). Finally, in BSO + BJ-treated cells, BJ was able to eliminate the effect of BSO and to maintain ALP levels at the values measured in untreated cells (Figure 2). 

**Figure 3:**
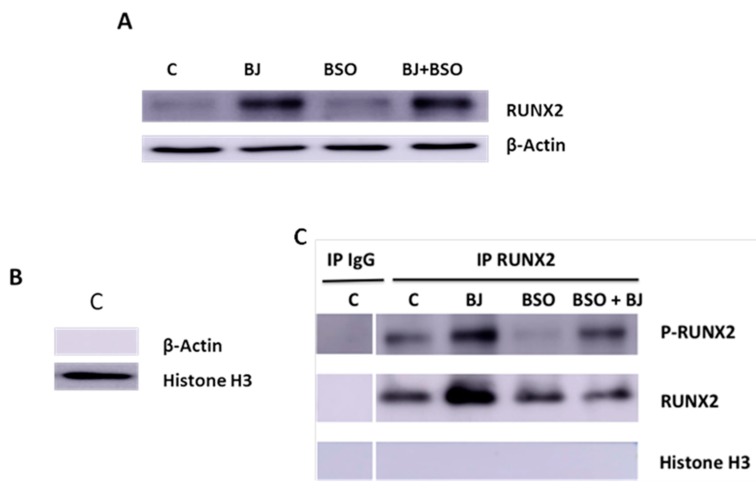
BJ effect on Runt-related transcription factor 2 (RUNX-2) expression and activation in BSO-treated and untreated SaOS-2 cells. RUNX-2 expression (**A**), nuclear fraction purification (**B**), and RUNX-2 activation (**C**) were detected in SaOS-2 cells treated or not with BJ at 15 μg·mL^−1^ total soluble polyphenols (TSP) or BSO or BSO + BJ for one day in growth medium (GM) and, subsequently, for six days in osteogenic medium (OM), in the presence or not of various treatments, as reported in Section 2. RUNX-2 expression was detected by Western blot analysis in whole cellular lysate (**A**), RUNX-2 activation was detected by phosphorylation in immunoprecipitates of nuclear extracts using the anti-RUNX-2 antibody (**C**), and β-actin and histone H3 were detected in nuclear extract (**B**). RUNX-2, p-RUNX-2, β-actin, and histone H3 were revealed with anti-RUNX-2, anti-p-tyrosine proteins, anti-β-actin, or anti-histone H3, respectively. The blots are representative of three experiments.

Subsequently, under the same experimental conditions, the expression and activation of RUNX-2 by tyrosine phosphorylation were studied six days after differentiation in SaOS-2 cell lysates and in nuclear extracts by Western blot analysis (Figure 3A–C). 

RUNX-2 is an important transcription factor for the activation of osteoblast differentiation and for regulation of bone remodeling, and it is involved in many bone diseases [44,45]. Figure 3A shows that BJ containing 15 μg∙mL^−1^ TSP was able to upregulate RUNX-2 levels, whereas no change was detected in cells treated with BSO only. RUNX-2 activation was evaluated by immunoprecipitation of equal amounts of nuclear proteins using anti-RUNX-2 antibody. The purification of the nuclear fraction was observed in control cells by Western blot, which revealed the presence of the histone H3 protein and not β-actin (Figure 3B). Figure 3C shows the absence of RUNX-2 or P-RUNX-2 or histone H3 bands in Western blot analysis of immunoprecipitates performed with IgG (negative control) in control cells; unlike RUNX-2 or P-RUNX-2 bands, no Histone H3 bands were detected after immunoprecipitation with anti-RUNX-2 (Figure 3). These data demonstrate the absence of non-specific bands under these experimental conditions. Figure 3C also shows that BJ containing 15 μg∙mL^−1^ TSP increased RUNX-2 nuclear levels and its phosphorylation as compared to control. On the other hand, decreases in RUNX-2 phosphorylation and no change in its nuclear levels were observed in cells treated with BSO, whereas RUNX-2 phosphorylation appeared to be restored by simultaneous treatment with BJ. Overall, these findings indicate that BJ was able to increase RUNX-2 and ALP activation, in normal redox state conditions, as well as prevent the inhibition of these factors in BSO-induced oxidative stress. 

### 3.4. BJ Effect on the Mineralization Process in SaOS-2 Cells in the Presence or Not of BSO-Induced Oxidative Stress

**Figure 4:**
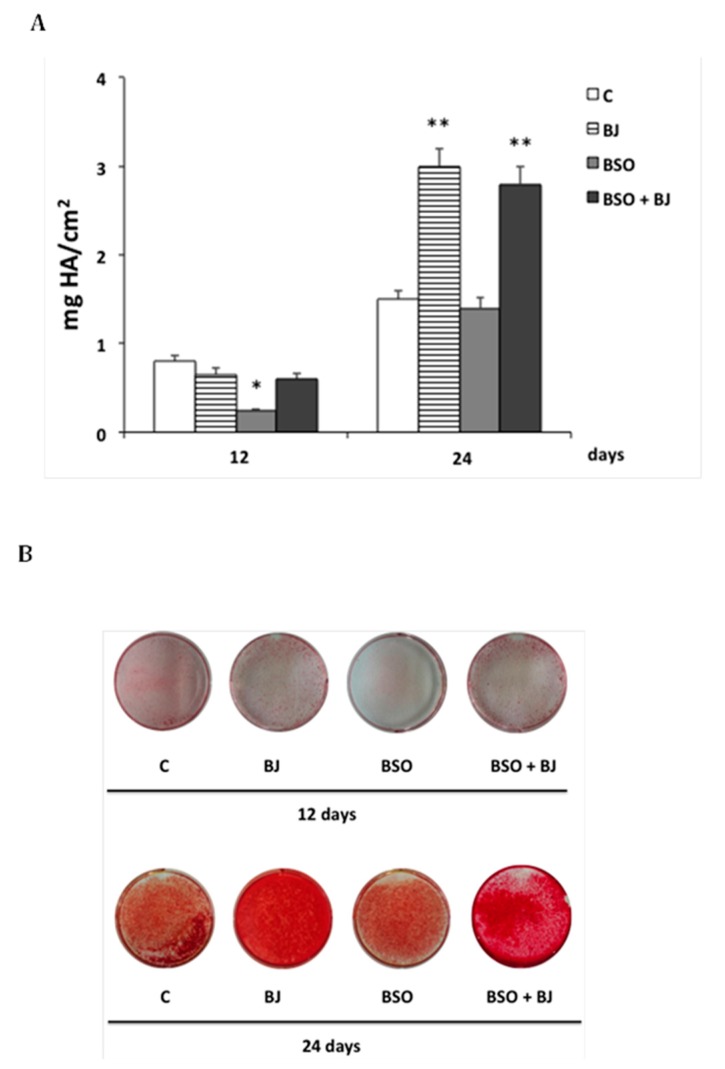
BJ effect on the mineralization process during differentiation of BSO-treated SaOS-2 cells. Calcium content, measured by Alizarin Red S assay, was detected in SaOS-2 cells treated or untreated with BJ, containing 15 μg·mL^−1^ total soluble polyphenols (TSP), or BSO or BSO + BJ for one day in growth medium (GM) and, subsequently, treated or untreated (C; control) for 12 or 24 days in osteogenic medium (OM), as reported in Section 2. Calcium content is expressed as mg hydroxyapatite (HA) per cm^2^ and values are means ± SEM of three independent experiments (**A**); * *p* ≤ 0.05, ** *p* ≤ 0.001 compared to the respective C cells. Representative images of calcium content in 12-well plates (**B**).

The mineralization process was studied in cells treated or not (control) with BJ containing 15 μg∙mL^−1^ TSP or with BSO or BJ + BSO. Calcium deposition was measured 12 and 24 days after the induction of differentiation by staining with Alizarin Red S (Figure 4A). 

Figure 4A,B show that BSO-induced oxidative stress in the initial phase of differentiation remarkably decreased the osteogenic activity after 12 days (by about 70%) as compared to control. This effect was prevented in BJ + BSO-treated cells, but no change in calcium deposition was observed in BJ-treated cells in this early phase as compared to control (Figure 4A,B). On the other hand, after 24 days, long-term activation of the mineralization process (of about 100%) was found in BJ- and BSO + BJ-treated cells, and no change was observed in BSO-treated cells as compared to control (Figure 4A,B). These data show that BJ phytochemicals prevent the anti-osteogenic effect of BSO-induced oxidative stress, and they are able to activate the mineralization process, even if this occurs later in time. 

### 3.5. Involvement of SIRT1 on BJ-Induced Activation of Osteogenic Differentiation and Mineralization Process in SaOS-2 Cells 

**Figure 5:**
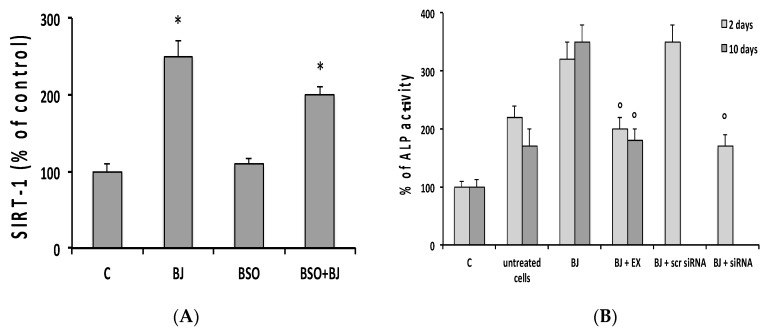
BJ effect on sirtuin type 1 (SIRT1) expression and SIRT1 inhibition role in BJ-induced ALP activation during differentiation of SaOS-2 cells. SIRT-1 expression (**A**) and ALP activity (**B**) were detected in SaOS-2 cells treated or not with BJ at 15 μg·mL^−1^ total soluble polyphenols (TSP) or BSO, BSO + BJ, or BJ + EX527, for one day in growth medium (GM) and, subsequently, in osteogenic medium (OM), in the presence or not of various treatments, as reported in Section 2. SIRT1 expression was detected after six days in OM by ELISA kit according to the manufacturer’s instructions, and levels are expressed as a percentage of the C values (A). ALP activity was also detected after two and 10 days in GM (control, C) and OM, as well as in transfected SIRT1 small interfering RNA (siRNA) or Scr RNA cells after two days in OM. SIRT1 expression and ALP activity values are expressed as a percentage of the respective C values, and they are the means ± SEM of three independent experiments; * *p* ≤ 0.05 compared to C cells; ^○^ *p* ≤ 0.05 compared to BJ-treated cells.

We subsequently evaluated the involvement of sirtuin type 1 (SIRT1), a class III histone deacetylase, in BJ-induced activation of osteogenic factors and the mineralization process, which occurred in the presence of a normal intracellular redox state. A possible role of SIRT1 with regard to the antioxidant activity of BJ was also studied. Indeed, SIRT1 is related to the regulation of osteogenic differentiation of tendon and mesenchymal stem cells [46,47], and it is a positive regulator of RUNX-2 [47,48]. Moreover, this enzyme is activated in mammals by dietary blueberry [49,50] and in osteocytes by BJ [33]. Initially, the BJ effect on SIRT1 levels in SaOS-2 cells in the presence or not of BSO-induced oxidative stress was studied. Figure 5A shows that BJ significantly increased SIRT1 expression as compared to control six days after differentiation, and a similar increase was also observed in BSO + BJ-treated cells, whereas no change in SIRT1 levels was detected in BSO-treated cells. 

Subsequently, the role of SIRT1 in BJ activation of osteoblast differentiation and mineralization was determined. To evaluate this, SIRT1 expression and activity were reduced using cells transfected with a specific SIRT1 siRNA or treated with EX527, a specific inhibitor of SIRT1, as reported in Section 2. Figure 5B reports that the downregulation of both SIRT1 and EX527, at the concentration able to inhibit SIRT1 activity [33], removed the activating effect of BJ on ALP activity two days after differentiation, and this effect was also observed at 10 days in EX527-treated cells. 

**Figure 6:**
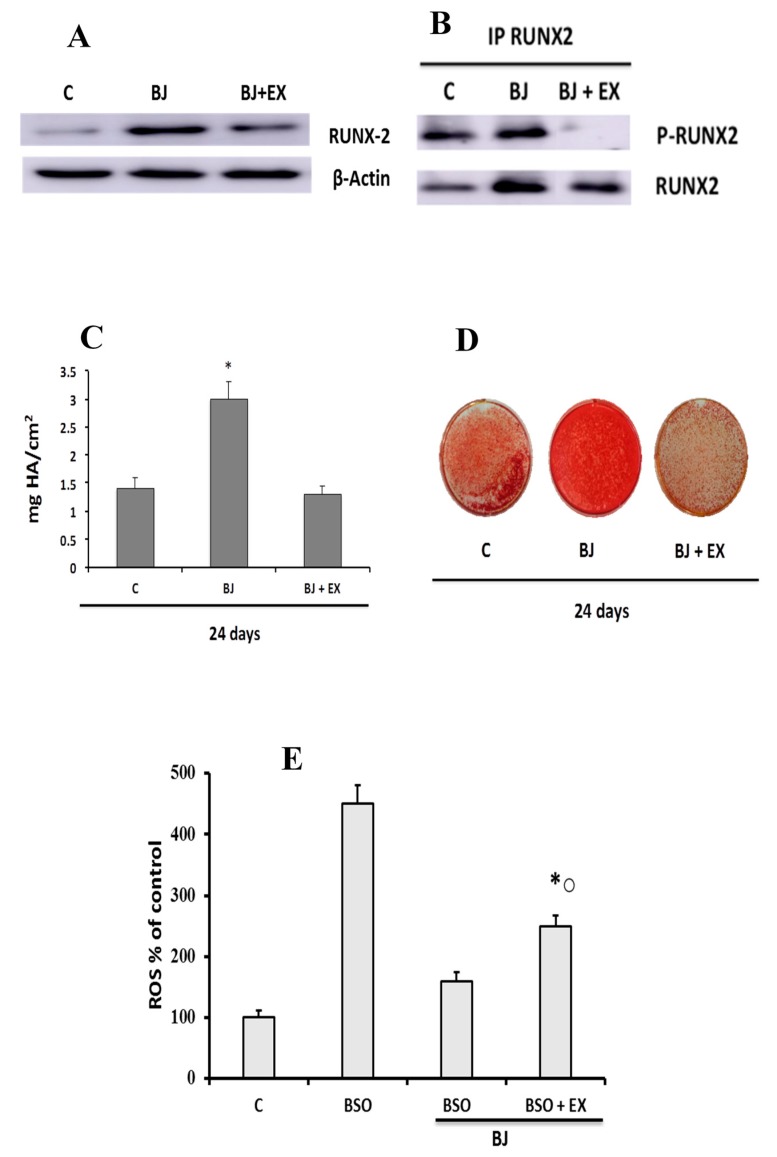
Effect of SIRT1 inhibition on BJ activation of RUNX-2 and the mineralization process, as well as on BJ antioxidant action during differentiation of SaOS-2 cells. RUNX-2 expression (**A**), RUNX-2 activation (**B**), calcium content (**C**,**D**), and ROS levels (**E**) were detected in SaOS-2 cells treated or not with BJ at 15 μg·mL^−1^ total soluble polyphenols (TSP) or BSO, BSO + BJ, BJ + EX527, or BJ + BSO + EX527, for one day in growth medium (GM) and, subsequently, in osteogenic medium (OM), in the presence or not of various treatments, as reported in Section 2. RUNX-2 expression was detected after six days in OM by Western blot analysis of whole cellular lysates (**A**), and RUNX-2 activation was detected by phosphorylation in immunoprecipitates of nuclear extracts using the anti-RUNX-2 antibody (**B**). RUNX-2, p-RUNX-2, and β-actin were revealed with anti-RUNX-2, anti-p-tyrosine proteins, and anti-β-actin, respectively. The blots are representative of three experiments. Calcium content was measured after 24 days in OM, and it is expressed as mg hydroxyapatite per cm^2^ (**C**). Representative images of calcium content in 12-well plates (**D**). ROS data are expressed as a percentage of the C values (**E**). The values are means ± SEM of three independent experiments; * *p* ≤ 0.05 compared to the respective C cells; ^○^ *p* ≤ 0.05 compared to BSO + BJ-treated cells.

SIRT1 involvement in BJ-induced RUNX-2 expression and activation was also studied in whole cellular lysates and in nuclear immunoprecipitates (Figure 6A,B). 

EX527 treatment partially prevented RUNX-2 expression (Figure 6A), as well as RUNX-2 activation (Figure 6B) induced by BJ six days after differentiation. The possible involvement of SIRT1 in long-term activation of the mineralization process induced by BJ is shown in Figure 6C,D. Finally, we evaluated the effect of SIRT1 on ROS levels in cells treated with BSO + BJ, considering that the antioxidant action of BJ could also be mediated by SIRT1. Figure 6E shows that BJ antioxidant action may only partially be mediated by SIRT1. 

## 4. Discussion

This study reports new data on the role of BJ phytochemicals and polyphenolic antioxidants in the activation of osteogenic factors and in the induction of the mineralization process in the presence or not of oxidative stress induced by GSH depletion. In particular, the polyphenolic content of the juice was characterized, and it was shown that BJ performs an important antioxidant action and protects from damage induced by oxidative stress, as well as upregulates factors such as ALP and RUNX-2, related to differentiation and the mineralization process, in normal intracellular redox state conditions. The involvement of SIRT1 in these events was also demonstrated. 

In this study, the effect of BJ containing certain amounts of TSP was evaluated in GSH-depleted SaOS-2 cells, an in vitro condition that mimics what happens in vivo in the bone environment in the presence of oxidative stress due to microdamage and/or estrogen deficiency, [4,5,8,22,51]. Indeed, GSH is involved in osteoblast and osteoclast differentiation and, together with other thiol antioxidants, it may play a crucial role in estrogen deficiency-associated bone loss [1,22,51,52]. In fact, some data show that bone loss due to a lack of estrogen is related to the lowered thiol antioxidants in osteoclasts, and this activates osteoclastogenic signals which induce ROS-enhanced expression of cytokines promoting osteoclastic bone resorption [51]. 

We used BJ given that BBs are commercialized in different ways, mainly as fresh or frozen products, also in addition to juices or dry extracts. However, the drying process and treatment with solvents (i.e., for the production of dry extracts) might partially destroy anthocyanins and their antioxidant effects [53], and the anthocyanins seem to be more stable over time in a juice with acidic pH than in a dry extract [53]. The results obtained from the qualitative and quantitative analysis of TSP in BJ show that the main polyphenolic component was represented by anthocyanins belonging to the flavonoid family, which were present mainly as glucosides, galactosides, and arabinosides; these data are similar to those found in the literature [42,54]. Moreover, TSP content in BJ obtained by HPLC-PDA was very similar to the value obtained with the Folin–Ciocalteu method, indicating that quantification via this spectrometric method is feasible and realistic.

Some studies demonstrated that many BB polyphenols are bioavailable; in fact, after various processes of ingestion, they were found in the plasma [36,54,55]. It was also demonstrated that anthocyanins or cyanidins, after oral administration, can be absorbed in intact form as glycosides and/or aglycones [34,35,55]. Moreover, even if BB polyphenols undergo complex metabolic modifications, their derivatives have the same functional characteristics [34,36]. The flavonoids and anthocyanins present in BB have strong antioxidant capacity [24,31,32,54], and these, along with the other polyphenol compounds and their derivatives, favor the formation of bone mass [18,19,20,30,54]. 

The data of this study demonstrate that TSPs, together with other phytochemicals contained in BJ, are able to prevent BSO-induced oxidative stress, and the results partly correlate with what was previously obtained with thiol antioxidants, such as GSH and *N*-acetyl cysteine, in SaOS-2 cells under similar conditions of oxidative stress [13]. The BJ antioxidant effect in SaOS-2 cells was obtained with the same concentrations of TSP used in osteocytes in which oxidative stress was induced by starvation [33], and ROS reduction was achieved by BJ treatment very quickly both in osteocytes and in SaOS-2 cells [13,33], similarly to thiol antioxidants [6]. Therefore, thanks to their antioxidant action, BJ phytocompounds may effectively contribute to preventing and/or eliminating oxidative stress damage present in bone pathologies, particularly in osteoporosis, as reported in the literature for various polyphenols or their derivatives [18,24,25,30]. Our results support these data; in fact, the initial stimuli that induce osteogenic activity in SaOS-2 cells are sensitive to changes in the oxidative state. Indeed, both ALP and RUNX-2 are important markers related to the activation of the first phase of the osteoblast differentiation process and to the subsequent induction of calcium and matrix deposition by differentiated osteoblasts [13,37,43,44]. In fact, we observed a strong initial increase of ALP activity that subsequently remained high and then decreased, indicating the achievement of a high degree of differentiation, as also previously observed [13]. Both ALP activity and RUNX-2 expression and activation significantly decreased in the presence of BSO-induced oxidative stress, as well as the mineralization process. These events are efficiently prevented by BJ antioxidant action.

The results of this study also show that BJ treatment in the presence of normal ROS levels has a remarkable non-redox-regulated osteogenic action that occurs through a significant upregulation of ALP and RUNX-2 activity. It is worthy to note that BJ maintains ALP activity levels higher than those in untreated cells for a long time, and this seems to be related to the activation of the mineralization process that is evident only in the late phase of this event. Finally, the similar and high levels of calcium, obtained in cells treated with BSO + BJ or with BJ alone after 24 days, show that the elimination of the oxidative state in the initial phase of the differentiation allows BJ to perform the long-term activation of this process. Indeed, modulation of ALP and RUNX-2 activity in BJ-treated SaOS-2 cells, in the presence or not of BSO-induced oxidative stress, is similar to that previously observed in these cells treated with thiol antioxidants, even if the effect of the latter on the mineralization process became evident more quickly [13]. These differences may be due to the different chemical characteristics and action mechanisms of the antioxidants. However, in both studies, thiol and non-thiol antioxidants regulated the osteogenic activity of osteoblasts through mechanisms unrelated to their antioxidant activity. In fact, the activating effect of BJ or of thiol antioxidants on the mineralization process occurred in cells in which there was no alteration in the redox state. It is worthy to note that the BJ effects on osteogenic factors were found using TSP concentrations similar to those used to demonstrate the osteogenic activity of the polyphenolic component of dried plums [56]. In fact, these antioxidants restore the TNFα-induced suppression of ALP activity and upregulate RUNX-2 expression, influencing mineralized matrix formation under normal and inflammatory conditions [56]. Moreover, our data agree with the increased expression of RUNX-2 and ALP in pre-osteoblast cells treated with serum from BB-fed rats and with the subsequent increase in osteoblast activity and bone formation [57]. These data are also related to the ability of BB-rich diets administered in young female rats to prevent bone loss in ovariectomized adult female rats [19,20,24,30], where the effects of dietary BB appear similar to those of estrogens [57]. In fact, the polyphenols seem to interact with the estrogen receptors and induce their effects through redox-independent factors and signaling pathways related to the regulation of bone cell activity [36,57].

Moreover, many data suggest that anthocyanins, in addition to their antioxidant activity, can also have other beneficial health effects [20,24,25,26]. This may agree with the possible involvement of SIRT1 in ALP and RUNX-2 activation, as well as the increase in calcium deposition, due to BJ treatment in the presence or not of a normal redox state. Indeed, SIRT1 levels did not change in the presence of BSO-induced oxidative stress, indicating that SIRT1 expression does not seem to be a redox-regulated mechanism. BJ was effectively able to upregulate SIRT1 expression in BSO-treated and untreated cells, and BJ antioxidant action was partly related to SIRT1 activity. Previously, we similarly demonstrated a significant relationship between BJ phytocompounds and molecular events related to apoptosis and expression of osteoclastogenic factors induced by oxidative stress and SIRT1 activation [33]. Indeed, dietary BBs increase SIRT1 levels in mammals [49,50], and SIRT1 overexpression is also related to the inhibition of osteoclastogenic factors [58,59]. Moreover, SIRT1 activity promotes osteogenic differentiation of mesenchymal stem cells and activation of RUNX-2 [46,47,48]. Previously, we also demonstrated that SIRT1 activity contributes, in part, to the BJ protective effect in MSCs against cytotoxicity due to oxidative damage [33]. Therefore, the possible role of SIRT1 can explain, at the molecular level, the positive action of TSP and/or other phytocompounds contained in BJ on the osteogenic activity and the mineralization process of osteoblasts, although other experiments will have to be performed to validate SIRT1’s involvement in these events. 

The activation of osteogenic factors and mineralization due to BJ treatment is also in agreement with the increase in bone mass found in young subjects fed with diets rich in blueberries or fruits rich in antioxidant phytochemicals [17,20,24,60,61]. It was also shown that BBs stimulate the growth of bone in growing rats, and this appears to be due to polyphenols and their metabolic derivatives [18,62]. Indeed, phenolic acid derivatives present in the diet promote the differentiation of osteoblasts and bone growth in young mice [63]. Some studies demonstrated that daily consumption of these compounds may be important in increasing the bone mass peak [20,60,64], and this is an independent predictor of increased bone mass in early pubertal children [61]. 

## 5. Conclusions

The present study demonstrated, in GSH-depleted SaOS-2 cells, that TSPs, together with other phytocompounds contained in BJ, are able to prevent early oxidative stress-induced inhibition of osteogenic differentiation and the mineralization process. This can occur in vivo in estrogen deficiency, as well as in aging and inflammatory diseases in which the loss of antioxidants leads to accelerated bone loss and, thus, to osteoporosis or osteopenia. It was also shown that the effect of BJ is not only due to its protective antioxidant activity, but also due to its ability to modulate signals that upregulate the expression and activity of osteogenic factors which are related to bone remodeling and bone formation. In fact, the increased expression of SIRT1 seems to be related to the osteogenic action of BJ, and these findings confirm that this enzyme may be considered a possible target for anti-resorptive drugs and for anabolic treatments for osteoporosis [58,59]. Finally, qualitative and quantitative analysis of the BJ soluble polyphenolic component showed the prevalence and high presence of anthocyanins. Overall, the results of this study, together with those previously obtained on osteocytes, may contribute to explain, at a cellular and molecular level, the beneficial effects of BBs on bone metabolism. In particular, they suggest that BJ effects are mainly related to TSP; in fact, polyphenols activate osteoblast function and inhibit osteoclast differentiation, thereby promoting bone growth [18,19,20,26,54,62]. Indeed, data obtained in animal studies showed the anabolic effects of BB in bone, and they suggested BB as a useful supplement for the prevention and/or management of osteoporosis and osteopenia [18,19,26,30,62]. In fact, anti-resorption drugs are effective in reducing bone mass loss and osteoclast activity, while they are also associated with limitations and side effects [28,65], as they do not restore a normal bone remodeling process [1,28]. Therefore, there is a growing demand for natural substances that can support medical therapy to reduce bone loss and restore normal bone metabolism. Future researches in vivo and in vitro are needed to better elucidate the molecular mechanisms underlying the action of a complex mixture of BB polyphenols on bone repair and formation processes in osteoporosis or bone inflammatory diseases related to oxidative stress. 

## Figures and Tables

**Table 1:** Identified polyphenols and total content of each class of constituents in blueberry juice.

Polyphenol Group	Identified Constituents	Total Polyphenols Expressed as Gallic Acid (mg ± SD/100 mL)
*Anthocyanins*	dephinidin 3-*O*-galactoside	
	delphinidin 3-*O*-glucoside	
	cyanidin 3-*O*-galactoside	
	delphinidin 3-*O*-arabinoside	
	cyanidin 3-*O*-glucoside	
	petunidin 3-*O*-galactoside	
	petunidin 3-*O*-glucoside	
	cyanidin 3-*O*-arabinoside	
	peonidin 3-*O*-galactoside	
	petunidin 3-*O*-arabinoside	
	malvidin 3-*O*-galactoside	
	peonidin 3-*O*-glucoside	
	malvidin 3-*O*-glucoside	
	malvidin 3-*O*-arabinoside	
**Total anthocyanins**		107.0 ± 9.3
*Flavonols*	quercetin 3-*O*-arabinoside	
	quercetin 3-*O*-galactoside	
	quercetin 3-*O*-glucoside	
	myricetin 3-*O*-glucoside	
	myricetin 3-*O*-galactoside	
**Total flavonols**		2.9 ± 0.9
*Flavan-3-ols*	catechin	
	epicatechin	
**Total flavanols**		5.8 ± 1.4
*Hydroxybenzoic acids*	ellagic acid	12.5 ± 2.4
*Hydroxycinnamic acids*	chlorogenic acid	30.6 ± 6.3

**Table 2:** Effect of butionine sulfoximine (BSO) and blueberry juice (BJ) on cell viability.

Days	1	2
Control	100 ± 8	100 ± 11
BSO	80 ± 10	83 ± 8
BJ	90 ± 8	85 ± 10
BSO + BJ	79 ± 9	80 ± 7

Viability was measured using the CellTiter-Glo Luminescent Cell Viability Assay in cells treated or not (control) with 40 μM BSO and/or BJ containing 15 μg·mL^−1^ of total soluble polyphenols. The data expressed as a percentage of the respective controls are means ± standard error of the mean (SEM) of four independent experiments.
